# Subconcussion revealed by sound processing in the brain

**DOI:** 10.1249/esm.0000000000000011

**Published:** 2023-06-28

**Authors:** Nina Kraus, Danielle Colegrove, Rembrandt Otto-Meyer, Silvia Bonacina, Trent Nicol, Jenna Cunningham, Jennifer Krizman

**Affiliations:** 1 Department of Communication Science and Disorders, Northwestern University, Evanston, IL, USA;; 2 Department of Neurobiology, Northwestern University, Evanston, IL, USA;; 3 Department of Otolaryngology, Northwestern University, Chicago, IL, USA; 4 Department of Sports Medicine, Northwestern University, Evanston, IL, USA

**Keywords:** Concussion, Subconcussion, Collegiate athletics, Contact sports, Auditory processing, Frequency-following response

## Abstract

**Introduction/Purpose:**

We tested the hypothesis that an objective measure of auditory processing reveals a history of head trauma that does not meet the clinical definition of concussion.

**Methods:**

Division I collegiate student-athletes (*n* = 709) across 19 sports were divided into groups, based on their sport, using prevailing classifications of “contact” (317 males, 212 females) and “noncontact” (58 males, 122 females). Participants were evaluated using the frequency-following response (FFR) to speech. The amplitude of FFR activity in a frequency band corresponding to the fundamental frequency (F0)—the voice pitch—of the speech stimulus, an outcome reduced in individuals with concussions, was critically examined.

**Results:**

We found main effects of contact level and sex. The FFR-F0 was smaller in contact athletes than noncontact athletes and larger in females than males. There was a contact by sex interaction, with the FFR-F0 of males in the contact group being smaller than the three other groups. Secondary analyses found a correlation between FFR-F0 and length of participation in contact sports in male athletes.

**Conclusion:**

These findings suggest that the disruption of sensory processing in the brain can be observed in individuals without a concussion but whose sport features regular physical contact. This evidence identifies sound processing in the brain as an objective marker of subconcussion in athletes.

## INTRODUCTION

Auditory processing, which is not merely hearing but also making sense of sound, is a newly recognized domain affected by concussions or mild traumatic brain injuries. While hearing acuity is typically unaffected by concussions, both behavioral and physiological measures of auditory processing, including in experimental animal models, indicate auditory processing impairment often accompanies a concussion (1–16).

An objective auditory physiological measure that is gaining purchase as an indicator of concussion is the frequency-following response (FFR) to speech. A chief outcome measure, the frequency-specific response to the fundamental frequency (F0) of speech—the voice pitch—is diminished in many individuals who experience an active concussion and is indicative, in recovered subjects, of past concussions (12,14,15). Because FFR is collected passively (i.e., it requires no input from the person under test), it is well positioned as a metric to augment concussion assessment, monitoring, and management, which otherwise are based on self-reported symptoms. Here, we investigated the FFR-F0 in healthy student-athletes with respect to the contact levels of the sports they practice.

We hypothesize a diminished FFR-F0 is a consequence of repeated contact that occurs over a lifetime of sports participation. We therefore predicted that a reduction in FFR-F0 would be measurable in athletes who compete in contact/collision sports (e.g., American football, lacrosse) or limited-contact sports (e.g., baseball, diving). Athletes in these sports engage in activities where collisions (with others or with the ground or equipment) occur to varying degrees. These subconcussive events rarely or never occur in the noncontact sports (e.g., swimming, tennis), which constituted our second group. We predicted a reduction in the FFR-F0 in participants of contact sports compared with participants of noncontact sports, representing a precursor to the response diminution that accompanies a concussion. Cumulative head injuries throughout an athlete’s career may lead to brain damage, a link supported by evidence of mild cognitive impairment and dementia in former contact-sport athletes (17,18). Results aligned with our predictions might represent an avenue for monitoring the impact of subconcussion in active athletes.

We collected FFR to the speech syllable “da” in 709 student-athletes at a Midwestern U.S. university whose sports teams are categorized as Division I by the National Collegiate Athletic Association (NCAA). We subdivided the athletes based on the nature of the sport they participated in, namely, contact and noncontact. The representation of males and females was balanced overall, but contact-level group membership was somewhat skewed because of the unbalanced participation of males and females in the two contact categories.

## METHODS

### Participants

We collected FFR in 709 (334 females) NCAA Division I student-athletes recruited from a Midwestern U.S. university (mean ± standard deviation (SD) age = 19.4 ± 1.2 yr). Table 1 shows the *n*s in each of our categories: contact/noncontact, male/female. This data set is part of a larger longitudinal data set where we collect FFR at least annually for all student-athletes until graduation; the data reported here come from the first test point collected. The design does not restrict initial enrollment to first-year students; consequently, the cohort comprises participants ranging from 17.5 to 22.0 yr. To verify normal hearing before FFR testing, all participants were screened with distortion-product otoacoustic emissions. For this study, we used the guidelines from Family Practice Notebook to arrive at the following categorization for the 19 Division I sports at Northwestern University (19): contact (includes limited contact), including men’s/women’s (M/W) basketball, M baseball, M/W dive, W field hockey, M football, W lacrosse, M/W soccer, W softball, W volleyball, and M wrestling; and noncontact, including W cross country, W fencing, M/W golf, M/W swim, and M/W tennis. All procedures were approved by the Northwestern University Institutional Review Board (approval no. STU00202670). Participants provided informed written consent to participate and were compensated monetarily for their time.

### FFR collection methods

Detailed FFR collection methods for our ongoing research program have been provided elsewhere (20). Briefly, data were collected on a Navigator Pro (Natus Medical, Inc., Middleton, WI, USA). The stimulus was a synthesized “da” with a duration of 40 ms, presented via insert earphone to the right ear at 80 dBA peak sound pressure level (SPL) at a rate of 10.9/s in alternating polarities. Two repetitions comprising 3000 sweeps each were presented after a brief click auditory brainstem response recording (31.1/s; 80 dBA peak SPL) that ensured within-normal limits of hearing sensitivity and appropriate earphone placement. Responses were differentially recorded from vertex to right earlobe with forehead as ground at a 12-kHz sampling rate and filters from 0.1 to 2.0 kHz.

### FFR processing

Responses (19.5–44.2 ms poststimulus onset) were converted to a frequency domain. Specifically, the selected segment was Hanning ramped (2 ms on, 2 ms off), baselined to its mean amplitude to remove any direct-current component, and submitted to 4096-point fast Fourier transformation. Magnitudes from 75 to 175 Hz were averaged to form the F0 metric.

### Statistical Analyses

The FFR-F0 amplitudes were not normally distributed, so they were log-transformed to create a normally distributed data set. A univariate analysis of variance (ANOVA) was first performed to rule out group differences in background noise level (evaluated by the log-transformed root-mean-square amplitude of the neural activity in the silent interval between stimulus presentations). The contact-level groups did not differ in this factor, which is known to affect the FFR to speech (*P* = 0.11). The log-transformed FFR-F0 amplitudes were then submitted to a univariate ANOVA, with age and number of reported past concussions as covariates. These covariates were used because, in addition to FFR-F0 amplitude being sensitive to acute concussion state, it exposes a legacy of past concussions, and FFR response characteristics also change over the life span (15). Although there is very little change in the FFR-F0 over the fairly restricted age range of participants in the present study (21), the groups did differ in age (F_3, 705_ = 3.28, *P* = 0.02, *η*^2^_p_ = 0.014), with the noncontact males (19.7 ± 1.2 yr) being older than the contact (19.3 ± 1.1 yr, *P* = 0.01) and noncontact (19.2 ± 1.0 yr, *P* = 0.003) females, and a trending difference with the contact males (19.4 ± 1.1 yr, *P* = 0.06). Therefore, age was used as a covariate as a precaution. Pairwise *post hoc* comparisons were evaluated by Fisher’s least significant difference test. A secondary analysis looked at the correlation between FFR-F0 and years that a participant had been active in their sport.

## RESULTS

**Figure 1.**
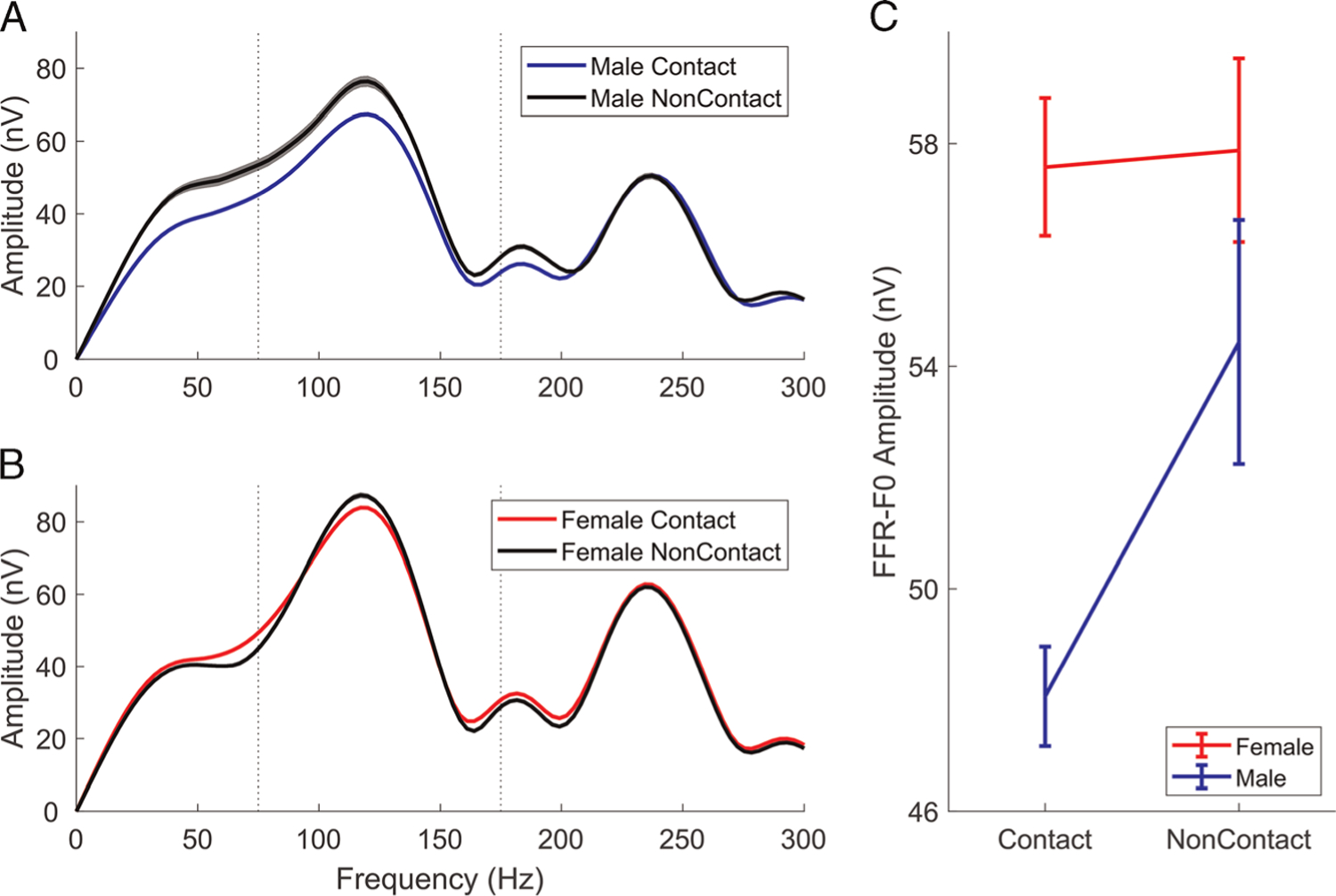
Frequency-following response (FFR) for male and female athletes. A. Average FFR spectra for the two contact groups for males. B. Average FFR spectra for the two contact groups for females. Both A and B have shaded regions indicating the mean ± 3 standard error (SE). A clear reduction in the male contact group can be seen at the fundamental frequency (F0) peak, centered on about 120 Hz. Female contact athletes do not show this reduction. C. Mean FFR-F0 ± 1 SE for each of the four groups. nV, nanovolts.

There was a main effect of group for F0 representation in the FFR (*F*_1,708_ = 7.27, *P* = 0.007, *η*^2^_p_ = 0.010), with the FFR-F0 smaller in the contact athletes (Fig. 1). Also, there was a main effect of sex (*F*_1,708_ = 15.60, *P* < 0.001, *η*^2^_p_ = 0.022), with female athletes having larger FFR-F0 than male athletes. There was an interaction between contact level and sex (*F*_1,708_ = 5.13, *P* = 0.02, *η*^2^_p_ = 0.007), with only males showing an FFR-F0 difference depending on contact level (*P* = 0.004) (Fig. 1). Female athletes had equivalent FFR-F0 amplitudes regardless of contact level (*P* = 0.89). See Table 2 for group mean amplitudes and Table 3 for pairwise *post hoc* results.

To better understand the FFR-F0 reduction we saw in male athletes, we consulted their subject histories for the reported number of years participating in a contact sport; these values ranged from 3 to 17 yr. Overall, there was a slight negative correlation (*r* = −0.15, *P* = 0.01) between log-transformed FFR-F0 and the number of years participating in a contact sport. The correlations were stronger in football (*r* = −0.25, *P* = 0.002) and soccer (*r* = −0.34, *P* = 0.10) players. No such relationships were found in female athletes.

## DISCUSSION

In this investigation into the effect of participation in contact sports on auditory brain function, we found a difference in sound processing in the brain between Division I collegiate contact and noncontact athletes. Specifically, the FFR-F0—a voice pitch cue essential for identifying individual players—was affected. This may help explain the overall reduction in the FFR-F0 observed in all collegiate athletes compared with nonathletes (22). Reduced FFR-F0 encoding is associated with concussion (12,14); the present results suggest that subconcussive events on the playing field, even when not serious enough to meet the threshold of a concussion diagnosis, may engender similar auditory processing disruptions as accompany concussions. Although the magnitude of FFR-F0 reduction is modest in the present study, it is likely a quantitative rather than qualitative difference because it mimics that seen in concussion. College undergraduate athletes are, relative to professional athletes, at the beginning of their careers. If an effect is observable at this stage, it may be a figurative canary in a coal mine. The long-term consequences of contact sports are increasingly under scrutiny, ranging from cognitive deficits in high school football players to high-profile cases of chronic traumatic encephalopathy discovered postmortem in professional athletes at soberingly high rates (23–29). The similarity of the auditory processing disruption found in the present study to that of concussion (both past and acute) suggests a potential role for FFR in the monitoring and early detection of subconcussive injuries that might lead to future cognitive difficulties and brain degeneration.

That female contact and noncontact athletes did not differ in FFR-F0 suggests a greater resilience in females to head trauma. The FFR-F0 is larger overall in females, an encoding advantage seen previously in adolescents and adults in the general population (30). We have also seen estrogen-mediated enhancements of other FFR components in experimental animals (31). Taken together, the present results may signal that the female brain has greater auditory processing reserves than the male brain. In addition to protecting the brain from milder sources of head trauma, greater processing reserves in females may also explain why females report greater symptom severity after a diagnosed concussion (32). That is, if the processing reserve can offset head trauma in females, the level of damage that females sustain to surpass the threshold for a concussion diagnosis would exceed that needed for males, resulting in greater symptoms in concussed females than males.

Despite the risks, athletic activity is one of the best things to do for health in many respects (33–35). Along with the well-known physical and mental health benefits, there are benefits for auditory health. A recent report found that during an auditory FFR session, student-athletes’ responses had a favorable signal-to-noise ratio primarily driven by a reduction in the level of background noise (20).

One potential limitation to our findings is the cross-sectional nature of our analyses. Because all athlete data points were collected in the same academic year, our data set ranged from first-year freshman athletes to fifth-year “redshirts.” We believe this confound, if it exists, would have reduced the likelihood of a contact group finding. Presumably an effect on auditory processing engendered by subconcussive contact would be the greatest in athletes who have participated the longest, and indeed we saw such a relationship. Yet even our snapshot, including a number of first-year student-athletes with less cumulative playing time, did not sufficiently dilute our findings in male athletes. The extent to which this range of experience had an impact, however, is mitigated by the fact that student-athletes playing collegiate sports at an elite level typically have quite a few years of high school or other extramural competition. Another factor that may have diluted our results is that many high school students play multiple sports of different contact levels. For example, a student might have played football in the autumn and tennis in the spring throughout high school, before settling on tennis as his collegiate athletic pursuit. This individual would have landed in our “noncontact” group. Again, the possible confound of complicated sports history would have worked against our findings because of potential category blurring. These potential confounds can be mitigated by collecting longitudinal data points annually to see how contact-sport patterns in the FFR evolve over the course of collegiate sport participation. This is in progress.

Additional refinement of the present and future longitudinal data sets might involve creating further subdivisions. For example, the single largest group among the 19 sports contributing to our data set is men’s football. There is a range of positions within football that vary in contact level. Offensive and defensive linemen, for example, collide with their opponents on nearly every snap, whereas other positions have less frequent contact. Digging into such a nuance will become possible as numbers increase, and we continue investigating auditory processing in student-athletes.

In conclusion, for the objective auditory processing metric reported here, male contact athletes have smaller responses than male noncontact athletes. This strengthens the idea that F0 encoding as a key signifier of concussion and positions it as a potential indicator of persistent minor head trauma that does not meet the clinical threshold of concussion in otherwise healthy athletes.

## Figures and Tables

**Table 1:** Participants by sex and contact group.

Group	Females	Males	Total
Contact	212	317	529
Noncontact	122	58	180
Total	334	375	709

**Table 2:** Group mean frequency-following response – fundamental frequency (FFR-F0) amplitude (standard deviation) in nanovolts.

Group	FFR-F0
Contact male	48.1 (16.0)
Contact female	57.6 (18.0)
Noncontact male	54.4 (16.7)
Noncontact female	57.9 (18.2)

**Table 3:** Pairwise *post hoc P* values (least significant difference) for the frequency-following response – fundamental frequency metric.

	Noncontact female	Noncontact male	Contact female
Contact male	**<0.001**	**0.004**	**<0.001**
Contact female	0.89	0.31	
Noncontact male	0.30		

## Data Availability

The data sets generated and/or analyzed during the current study are available from the corresponding author upon reasonable request.
